# Mouse Models Targeting Selenocysteine tRNA Expression for Elucidating the Role of Selenoproteins in Health and Development 

**DOI:** 10.3390/molecules14093509

**Published:** 2009-09-10

**Authors:** Bradley A. Carlson, Min-Hyuk Yoo, Petra A. Tsuji, Vadim N. Gladyshev, Dolph L. Hatfield

**Affiliations:** 1 Molecular Biology of Selenium Section, Laboratory of Cancer Prevention, Center for Cancer Research, National Cancer Institute, National Institutes of Health, Bethesda, MD 20892, USA;E-mails: yoom@mail.nih.gov (M-H.Y.); tsujipa@mail.nih.gov (P.A.T.); hatfield@mail.nih.gov (D.L.H.); 2 Cancer Prevention Fellowship Program, National Cancer Institute, National Institutes of Health, Bethesda, MD 20892, USA; 3 Nutritional Science Research Group, Division of Cancer Prevention, National Cancer Institute, National Institutes of Health, Bethesda, MD 20892, USA; 4 Department of Biochemistry and Redox Biology Center, University of Nebraska, Lincoln, NE 68588, USA; E-mail: vgladysh@unlnotes.unl.edu (V.N.G.)

**Keywords:** mouse models, selenium, selenocysteine tRNA, selenoproteins

## Abstract

Selenium (Se) deficiency has been known for many years to be associated with disease, impaired growth and a variety of other metabolic disorders in mammals. Only recently has the major role that Se-containing proteins, designated selenoproteins, play in many aspects of health and development begun to emerge. Se is incorporated into protein by way of the Se-containing amino acid, selenocysteine (Sec). The synthesis of selenoproteins is dependent on Sec tRNA for insertion of Sec, the 21^st^ amino acid in the genetic code, into protein. We have taken advantage of this dependency to modulate the expression of Sec tRNA that in turn modulates the expression of selenoproteins by generating transgenic, conditional knockout, transgenic/standard knockout and transgenic/conditional knockout mouse models, all of which involve the Sec tRNA gene, to elucidate the intracellular roles of this protein class.

## 1. Introduction

Selenium (Se) is an essential element in the diet of many life forms including humans and other mammals. Numerous health benefits and cellular functions have been attributed to this element, including roles in preventing cancer, heart disease and other cardiovascular and muscle disorders, delaying the aging process and the onset of AIDS in HIV-positive patients, inhibiting viral expression, and supporting mammalian development, male reproduction and immune function [1]. Selenoproteins have a major role in health and development which has only recently begun to be understood, due in large part to the targeted removal of individual selenoproteins or all selenoproteins in specific cells, tissues and organs of mice.

Se is incorporated into selenocysteine (Sec) in the biosynthesis of this amino acid that constitutes the 21^st^ naturally-occurring amino acid in the genetic code [2,3,4]. Sec biosynthesis, unlike any other known amino acid in eukaryotes, occurs on its tRNA, designated Sec tRNA^[Ser]Sec^ [5], and the biosynthetic pathway of Sec was only recently established in eukaryotes and archaea [6,7]. Once Sec is biosynthesized on its tRNA, this amino acid is co-translationally inserted into protein. There are 24 Se-containing protein (selenoprotein) genes in rodents and 25 in humans [8]. One of the unique properties of selenoproteins is that their synthesis is dependent on Sec tRNA^[Ser]Sec^ and, if the expression of this tRNA is altered, then the expression of the resulting class of selenoproteins is altered [9,10]. We have taken advantage of the fact that selenoprotein expression can be controlled by manipulating the expression of Sec tRNA^[Ser]Sec^ to generate a number of mouse models for elucidating the intracellular roles of selenoproteins [9,10,11,12], which is the subject of this review. 

It should be noted that numerous other studies have reported the targeted removal of individual selenoproteins which have elucidated their intracellular roles. For example, glutathione peroxidase 1 (GPx1) [13] and glutathione peroxidase 2 (GPx2) knockout mice [14] develop normally with little or no noticeable change in their phenotypes. However, a combined GPx1/GPx2 knockout mouse develops colitis at an early age [15]. GPx4, on the other hand, was shown to be essential for mouse development as GPx4 deficient mice die *in utero* at E7.5 [16]. Knockout of thioredoxin reductase 1 (TR1) [17] and thioredoxin reductase 3 (TR3) [18] have also been shown to be embryonic lethal. Deiodinase 1 (Dio1) [19] and deiodinase 2 (Dio2) knockout mice [20] appear healthy and reproduce normally. However, the Dio2 knockout mice have impaired auditory function, impaired thermogenesis and exhibit mild defects in brain function. A combined Dio1/Dio2 knockout mouse has a similar mild phenotype but exhibits a greater alteration of brain gene expression [21], while deiodinase 3 (Dio3) knockout mice have been shown to have reduced viability, fertility and growth retardation [22]. Selenoprotein P (SelP) knockout mice manifest neurological defects with ataxia and seizures [23,24], and recently a selenoprotein R (SelR) knockout mouse has been shown to be viable and appeared normal despite a tissue-dependent increase in oxidative stress [25]. A very thorough review covering the knockout of individual selenoproteins in mice has recently been published [26].

## 2. Mouse Models Involving the Sec tRNA^[Ser]Sec^ Gene (*Trsp*)

Removal of the Sec tRNA^[Ser]Sec^ gene (*Trsp*) from the mouse genome is embryonic lethal, demonstrating that the expression of selenoproteins is essential to the development of mammals [11,27]. Therefore, to study the intracellular roles of selenoproteins using Sec tRNA^[Ser]Sec^ as a tool to manipulate the expression of this Se-containing protein class, we have developed mouse models that encode (1) wild type or mutant transgenes [9], (2) a conditional knockout of *Trsp* [11], or (3) a combination of a standard knockout of *Trsp* and mutant or wild type transgenes or a conditional knockout of *Trsp* and mutant or wild type transgenes [10,28]. Each of these mouse models and their uses are further discussed below.

### 2.1. Trsp transgenic mouse models

Several transgenic mouse models have been generated with genomes encoding wild type or mutant Sec tRNA^[Ser]Sec^ transgenes that varied in the number of transgene copies and in the position of the mutation within the tRNA [9]. The site of the mutation in Sec tRNA^[Ser]Sec^ was at position 37 (A37G37; [9]) or at position 34 (T34A34; [28]). In the fully mature tRNA, position 37 normally has an isopentenyladenosine (i^6^A) and the base at position 34 is methylcarboxymethyl-5’-uridine (mcm^5^U) [29,30]. The number of transgene copies varied from two to as many as 40 with either the wild type or G37 mutant [9], while the maximum number of transgene copies with the position 34 (A34) mutant was less, and appeared not to exceed 12 in a wild type *Trsp* background or two in a Δ*Trsp* background [28]. The reason for the lower copy number of transgenes in A34 mutant is likely due to the fact that an A at position 34 in tRNA is converted to inosine which in turn decodes U/C/A in the 3’-position of the corresponding codeword (see [28] and references therein). Thus, Sec tRNA^[Ser]Sec^_T34__A34_ would be expected to decode the Sec codon, UGA, and the cysteine (Cys) codons, UCU/UCC. The subsequent misreading of the Cys codons inserting Sec would most certainly be deleterious to cellular function. 

**Figure 1:**
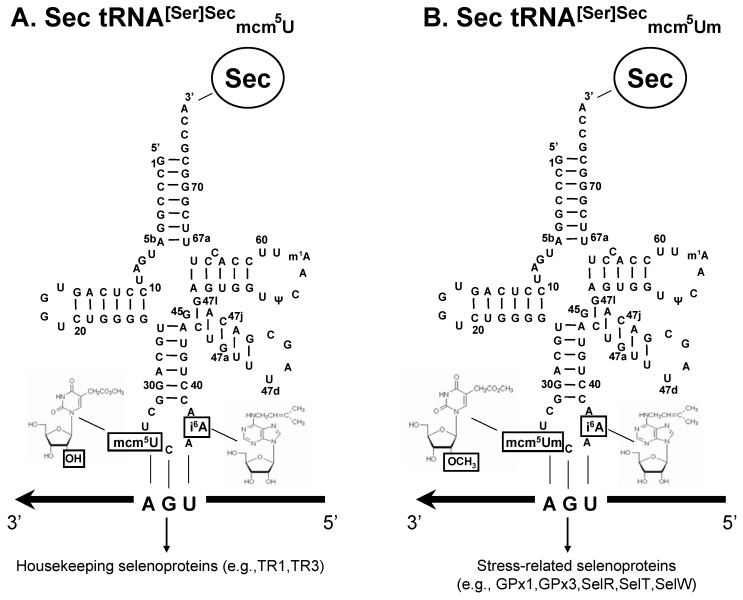
Primary structures of bovine liver (A) selenocysteyl-tRNA^[Ser]Sec^_mcm_^5^_U_ and (B) selenocysteyl-tRNA^[Ser]Sec^_mcm_^5^_Um_ shown in a cloverleaf model. Mammalian Sec tRNA^[Ser]Sec’^s are 90 nucleotides in length and have modified bases at positions 34 (mcm^5^U), 37 (mcm^5^Um; and although the modification at position 37 is often referred to as a modified base, Um34 is a methyl group addition to the 2’-position of the ribose and thus the modification results in a modified nucleoside), 55 (pseudouridine; ψ) and 58 (N1-methyladenosine, m^1^A). The structures of the two highly modified bases at positions 34 and 37, mcm^5^U and mcm^5^Um, respectively, are shown in the figure and Sec tRNA^[Ser]Sec^_mcm_^5^_U_ is responsible for synthesis of housekeeping selenoproteins and Sec tRNA^[Ser]Sec^_mcm_^5^_Um_ for stress-related selenoproteins. The bases at positions 34 and 37 have been mutated, T34A34 and A37G37, and the consequences of these mutations are that neither mutant tRNA can form Um34 resulting in a virtual loss in stress-related selenoprotein expression (see text for details.).

It should also be noted that the Sec tRNA^[Ser]Sec^ population in mammalian cells normally consists of two isoforms that differ from each other by a single 2’-*O*-methylribose at position 34, designated Um34 (see Figure 1; [29] and references therein). The synthesis of Um34 on the mcm^5^U base to form methylcarboxymethyl-5’-uridine-2’-*O*-hydroxmethylribose (mcm^5^Um) is the last step in the maturation of Sec tRNA^[Ser]Sec^ [30]. This is regarded as a highly specialized step in that its addition is dependent on the correct primary, secondary and tertiary structure of the tRNA [31]. Thus, both the A34 and G37 mutant tRNAs lack Um34 (see [28] and references therein). Um34 synthesis is also dependent on Se status wherein animals (or cells) maintained on a Se deficient diet (media) have low levels of mcm^5^Um-containing Sec tRNA^[Ser]Sec ^compared to mcm^5^U-containing Sec tRNA^[Ser]Sec^, while animals (or cells) maintained on an adequate or enriched Se diet (media) have higher levels of mcm^5^Um-containing Sec tRNA^[Ser]Sec^ compared to mcm^5^U-containing Sec tRNA^[Ser]Sec ^ [32]. The levels of the two isoforms correlate with the expression levels of the two subclasses of selenoproteins, stress-related (e.g., GPx1, glutathione peroxidase 3 (GPx3), SelR, selenoprotein T (SelT), selenoprotein W (SelW) and housekeeping (e.g., TR1, TR3) (see Figure 1 and [33] for review). Selenoproteins that are most responsive to Se status and the Sec tRNA^[Ser]Sec^ Um34 modification serve largely stress-related functions and they are not essential to the animal’s survival. On the other hand, selenoproteins that are less responsive to selenium status and the Sec tRNA^[Ser]Sec^ Um34 modification serve largely housekeeping functions and are essential to the animal’s survival. Stress-related selenoproteins, like the mcm^5^Um-containing Sec tRNA^[Ser]Sec^ isoform, are sensitive to Se status [29], and whether the amount of mcm^5^Um-containing Sec tRNA^[Ser]Sec^, which governs the expression of this selenoprotein subclass, is responsible for lack of synthesis of a stress-related selenoprotein that in turn renders the untranslated mRNA available for nonsense-mediated decay (NMD) [34,35] is not known. 

The transgenic mouse models involving G37 (i^6^A^-^)transgenes, their uses and the major findings in each of these studies are summarized in Table 1.

**Table 1:** G37 mutant *Trsp* transgenic mouse models.^a^

Transgene Number^b^	Model Description	Major Findings	Reference
2-4, 8-16, 20-40	Mice encode a mutant G37 transgene in all tissues and organs.	Levels of stress-related selenoproteins decreased in a protein and tissue specific manner in mice expressing a mutant G37 tRNA^[Ser]Sec^ isoform that also lacks Um34. GPx1 and TR3 were the most and least affected selenoproteins, while selenoprotein expression was most and least affected in the liver and testes, respectively. **First transgenic mouse generated encoding a tRNA transgene.**	[9]
40	Mice encode a mutant G37 transgene in all tissues and organs.	Enhanced skeletal muscle adaptation after exercise enhanced growth in the G37 mice that was completely blocked by inhibition of the mammalian target of rapamycin (mTOR) pathway. Muscles of transgenic mice exhibited increased site-specific phosphorylation on both Akt and p70 ribosomal S6 kinase before ablation.	[72]
40	Mice encode a mutant G37 transgene in all tissues and organs. Colon is targeted with azoxymethane.	Mice had more azoxymethane-induced aberrant crypt formation (a preneoplastic lesion for colon cancer). **First demonstration that selenoproteins reduce colon cancer incidence.**	[40]
20	Mice encode a mutant G37 transgene in all tissues and organs and a prostate cancer driving *C3(1)/Tag* transgene.	Mutant mice exhibited accelerated development of lesions associated with prostate cancer progression, implicating selenoproteins in cancer risk and raising the possibility that Se prevents cancer by modulating the levels of stress-related selenoproteins. **First demonstration that selenoproteins reduce prostate cancer incidence.**	[42]
40	Mice encode a mutant G37 transgene in all tissues and organs.	Mutant mice showed higher micronuclei formation than control mice in erythrocytes following exposure to X-rays.	[73]
40	Mice encode a mutant G37 transgene in all tissues and organs. Lung is targeted by administration of influenza virus.	At day 2 p.i., chemokine levels were greater in the G37 mice compared with wild type mice. Additionally, IFN-γ was higher at day 7 p.i. in the G37 mice and viral clearance slower. Despite these immune system changes, lung pathology was similar in G37 and wild type mice.	[74]

^a^The transgene in each case encoded a mutation at position 37 (AG) and lacks both isopentenyladenosine at position 37 and Um34 [31]; ^b^Number of transgenes carried by transgenic mice and 2-4, 8-16, or 20-40 designate whether the animal is heterozygous or homozygous.

Since wild type transgenic mice encoding as many as 20 extra copies of *Trsp* highly over-express Sec tRNA^[Ser]Sec^ with apparently little or no effect on selenoprotein synthesis, it appears that Sec tRNA^[Ser]Sec^ is not limiting in the expression of this protein class (reviewed in [33]). The transgenic mouse model encoding G37 transgenes was initially used to examine the effect of this mutant tRNA on selenoprotein expression [9]. Interestingly, selenoproteins were synthesized in a specific manner wherein the expression of stress-related selenoproteins was down-regulated (e.g., GPx1), while the expression of housekeeping selenoproteins was up-regulated (e.g., TR1). This alteration in selenoprotein synthesis was subsequently found to be due to the lack of expression of mcm^5^Um-containing Sec tRNA^[Ser]Sec^, as is further discussed below in subsections *2.3.* and *2.4.*

The effect of the G37 mutant tRNA on selenoprotein expression also occurred in a tissue specific manner wherein, for example, little or no change in selenoprotein expression occurred in testes, but dramatic changes in relative amounts of stress-related selenoproteins, i.e., GPx1, occurred in liver [9]. The tissue specificity of selenoprotein expression was due to the amounts of the Sec tRNA^[Ser]Sec^ population in the respective organ which is, for example, three to four times higher in testes than liver (see [33] for a review). 

The G37 mouse model has also been used for studying the role of selenoproteins in health (Table 1) and those studies that have shown an involvement of selenoproteins in cancer protection are of particular interest. It has been known for many years prior to these studies involving G37 mouse models that Se has a protective effect against certain forms of cancer [36,37], but it was not known whether selenoproteins or low molecular weight selenocompounds were responsible [38,39]. In an initial study, G37 transgenic and wild type mice were fed a Se deficient diet that was either supplemented or not supplemented with Se and the mice treated with azoxymethane, which is a specific colon carcinogen [40]. G37 transgenic mice had far greater azoxymethane-induced aberrant crypt formation than wild type controls and supplemental Se in the diets reduced the number of aberrant crypts in both transgenic and control mice. Since aberrant crypt formation is considered a preneoplastic lesion marker for colon cancer, the data provided strong evidence that a reduction in stress-related selenoproteins caused by the G37 mutant Sec tRNA^[Ser]Sec^ enhanced colon cancer incidence. The fact that transgenic and control mice also had higher amounts of Se in non-protein fractions of colon tissue indicating higher amounts of small molecular weight selenocompounds in this tissue suggested that non-protein Se-containing compounds also have a role in colon cancer prevention. This study was the first to demonstrate that both selenoproteins and low molecular weight selenocompounds have roles in cancer prevention [40]. Interestingly, a study examining the selenoproteins most affected in colon of mice fed a marginally Se-deficient (0.086 mg of Se/kg) diet was carried out showing that the levels of selenoproteins GPx1, W, H and M responded most significantly to the lower Se levels [41]. Several of these selenoproteins (e.g., GPx1, selenoprotein W and selenoprotein H) have also been shown to be sensitive to the levels of G37 mutant Sec tRNA^[Ser]Sec^ in transgenic mice [10,33]. Thus, these selenoproteins appear to be regulated by the amount of mcm^5^Um-containing Sec tRNA^[Ser]Sec^ and could possibly serve as biomarkers for assessing Se status.

The G37 mouse model has also been used to show that selenoproteins have a role in prostate cancer prevention [42]. These investigators crossed a homozygous G37 mutant transgenic mouse carrying 20 copies of the mutant transgene/allele with a homozygous *C3/Tag* transgenic mouse (*C3/Tag* is a prostate cancer driver gene) to assess prostate tumor formation in offspring using wild type *C3/Tag* mice as controls. The stress-related selenoprotein deficient C3/Tag mice manifested a significantly higher number of prostatic intraepithelial neoplasia (PIN) lesions associated with prostate cancer progression than the controls [42]. 

### 2.2. Trsp conditional knockout mouse models

As noted above, removal of *Trsp* in the mouse genome is embryonic lethal. Further examination of the effect of knocking out this gene, therefore, had to be carried out by conditionally targeting its removal from specific tissues and cells. We used *loxP-Cre* technology to generate a conditional knockout of *Trsp* [11]. As a result, various cells, tissues and organs have been targeted for *Trsp* removal as shown in Table 2. These studies show unequivocally that selenoproteins have a role in many parameters of development and disease prevention. 

For example, Se has been known for many years to have a role in boosting immune function [43,44]. However, the targeted removal of this protein class from T cells [45] and macrophage ([46]; B.A. Carlson, M.-H. Yoo, Y. Sano, A. Sengupta, J.Y. Kim, R. Irons, V.N. Gladyshev, D.L. Hatfield and J.M. Park [unpublished data]) demonstrated a role of selenoproteins in the function of the immune system. More specifically, the knockout of *Trsp* in T cells resulted in T cell dependent antibody responses, a reduced number of mature T cells and an oxidant hyperproduction that in turn suppressed T cell proliferation in response to T cell receptor stimulation [45]. The targeted removal of *Trsp* in macrophages and of the transcription factor *Nrf2* in the same mouse resulted in reduced viability, and an enhancement in oxidative stress and in the animal’s susceptibility to hydrogen peroxide compared to that which was observed by knocking out either single gene [46]. Targeting the removal of only *Trsp* in macrophage resulted in increased expression of genes involving oxidative stress and detoxification enzymes [46].

In another study that targeted the *Trsp* removal in macrophage, this cell type was found to manifest largely normal inflammatory responses, but selenoprotein loss had abnormal expression of extracellular matrix-related genes and a reduced migration of macrophages in a protein gel matrix (B.A. Carlson, M.-H. Yoo, Y. Sano, A. Sengupta, J.Y. Kim, R. Irons, V.N. Gladyshev, D.L. Hatfield and J.M. Park [unpublished data]). These studies on targeting removal of *Trsp* in T cells and macrophages provide strong evidence that Se status affects immune defense and tissue homeostasis mediated by selenoproteins, and furthermore, a role in trafficking of tissue macrophages. 

To elucidate the role of selenoproteins in neuronal function, the removal of floxed *Trsp* was targeted in neurons using Cre recombinase under the control of the neuronal-specific Tα1 antigen-promoter (E.K. Wirth, M. Conrad, J. Winterer, C. Wozny, S.B. Bharathi, C. Iserhot, B.A. Carlson, S. Roth, D. Schmitz, G.W. Bornkamm, M. Brielmeier, V. Coppola, L. Tessarollo, E. Pohl, L. Schomburg, J. Kohrle, D.L. Hatfield. and U. Schweizer [unpublished data]). Mice lacking the ability to synthesize selenoproteins in neuronal tissue lost postural control and developed seizure-like behavior. Interneurons in the cerebral cortex and hippocampus did not develop parvalbumin expression and extensive neuro-degeneration was observed in these two brain regions. Affected offspring lived about two weeks and, in addition, suffered from cerebellar hypoplasia with Purkinje cell death and decreased granule cell proliferation. Specific knockout of the GPx4 gene (*Gpx4*) manifested similar cerebellar and interneuron phenotypes that mice with total loss of selenoprotein expression exhibited illustrating the importance of selenoproteins, and specifically Gpx4*,* in neuronal development and function. 

**Table 2:** *Trsp* conditional knockout mouse models.

Cre Promoter	Targeted Organ or Tissue	Major Findings	Reference
*MMTV-Cre; Wap-Cre*	Mammary gland	**First description of the *Trsp* conditional knockout mouse.**	[11]
*Alb-Cre*	Liver	Death between 1 and 3 months of age due to severe hepatocellular degeneration and necrosis. Elevated GST levels [12]. Brain Se levels are maintained in the absence of liver-derived plasma SePP [75]. Hepatic Dio1 is not essential to maintain plasma thyroid hormone levels [76]. **Selenoproteins have a role in proper liver function.**	[12,75,76]
*TieTek2-Cre*	Endothelial cell	14.5 dpc embryos were smaller in size, more fragile, had a poorly developed vascular system, underdeveloped limbs and tails and heads. **Selenoproteins have a role in endothelial cell function.**	[53]
*MCK-Cre*	Heart and skeletal muscle	Died from acute myocardial failure day 12 after birth. **Selenoproteins have a role in preventing heart disease.**	[53]
*LysM-Cre*	Macrophage	Elevated oxidative stress and transcriptional induction of cytoprotective antioxidant and detoxification enzyme genes. Accumulation of ROS levels and impaired invasiveness. Altered expression of several extracellular matrix and fibrosis-associated genes. **Selenoproteins have a role in immune function.**	[46]; Carlson *et al*.^a^
*NPHS2-Cre*	Kidney	Loss of podocyte selenoproteins does not lead to increased oxidative stress or worsening nephropathy.	[77]
*LCK-Cre*	T cells	Decreased pools of mature T cells and a defect in T cell-dependent antibody responses. Antioxidant hyperproduction and thereby suppression of T cell proliferation in response to T cell receptor stimulation. **Selenoproteins have a role in immune function.**	[45]
*T* *α* *1 antigen-Cre*	Neuron specific	Enhanced neuronal excitation followed by massive neurodegeneration of the hippocampus. Cerebellar hypoplasia was associated with degeneration of Purkinje and granule cells. Cerebellar interneurons were essentially absent. **Selenoproteins have a role in neuronal function.**	Schweizer *et al*.^a^
*Col2a1-Cre*	Osteo-chondroprogenitor	Post-natal growth retardation, chondrodyplasia, chondronecrosis and delayed skeletal ossification characteristic of Kashin-Beck disease. **First model for Kashin-Beck disease.**	[48]
*K14-Cre*	Skin	Runt phenotype, premature death, alopecia along with a flaky and fragile skin, epidermal hyperplasia with disturbed hair cycle and an early regression of hair follicles. **Selenoproteins have a role in skin and hair follicle development.**	Sengupta *et al*.^a^

^a ^Unpublished data (see text).

The targeted removal of *Trsp* in epidermal tissue of the skin yielded mice with stunted growth and a shorter life span (mean life span was 10 days), flaky skin that was wrinkled and fragile, sparse hair wherein hair loss increased with age and reduced intradermal body fat (A. Sengupta, U.F. Lichi, B.A. Carlson, A.O. Ryscavage, V.N. Gladyshev, S.H. Yuspa, D.L. Hatfield [unpublished data]). Histological analysis of hair follicles revealed a decreased number of follicles with growth retardation, while histological analysis of epidermal tissue revealed moderate epidermal hyperplasia along with acute focal coagulative necrosis of the epidermis. The initiation of hair follicle formation appeared normal, but hair follicles underwent premature repression in knockout mice. Keratinocytes from the knockout mice were impaired in attachment and proliferation, though antioxidants like vitamin E improved their attachment and survival. These observations highlighted an essential role of selenoproteins in epidermal function including hair follicle morphogenesis and manifested a novel role of selenoproteins in skin function and development.

#### 2.2.1. Mouse models relating to human disease

It should also be noted that the studies summarized in Table 2 implicate a role of selenoproteins in human diseases. For example, Kashin-Beck disease, which is found in various regions of China and has been described in North Korea and Siberia, is an osteoarticular disease involving cartilage and is characterized by stunted growth, skeletal deformities and arthropathy of multiple joints [47]. The etiology of the disease has not been fully resolved, but Se and iodine deficiency, along with organic materials that apparently serve as toxins in the drinking water, appear to be involved. To assess whether a mouse model could be produced for Kashin-Beck disease, a Cre recombinase transgenic mouse line was generated that targeted *Trsp* removal in osteo-chondroprogenitors [48]. The resulting mutant mice suffered growth retardation, delayed skeletal ossification and a pronounced chondronecrosis of cartilages in various tissues. The mice appeared to phenotypically mimic numerous pathological features of Kashin-Beck disease providing further evidence that Se deficiency may be important in the development of this disease. 

Keshan disease is a congestive cardiomyopathy occurring in northeastern China and named after the county in which it was first observed [49]. The disease is caused by a dietary deficiency that originates from the scarcity of Se within the soil of the region, that in turn renders the local vegetation and livestock Se deficient and thus the local inhabitants. The disease has virtually been eradicated by supplementing the diets of the inhabitants with Se [50]. There is a cofactor associated with the disease that appeared to be coxsackie B virus. Beck and collaborators have provided a mouse model for Keshan disease wherein mice maintained on a Se sufficient diet and infected with coxsackie B3 virus were not affected and the virus remained non-virulent (see review in [51]). However, if the mice were maintained on a Se deficient diet and infected with the virus, the virus becomes virulent and the mice develop a cardiomyopathy. Once the virus becomes virulent, it is always virulent, and interestingly, there are six nucleotide changes between the virulent and non-virulent viral forms [51]. The virulent form appears to exist as a minor population among the non-virulent form and becomes dominant in mice that are Se deficient. 

We are only beginning to understand the role of selenoproteins in heart function and development, and their possible role in Keshan disease as well as other cardiovascular diseases. Mitochondrial thioredoxin reductase 3 (TR3) is highly expressed in heart and its specific knockout in cardiac tissue resulted in a fatal dilated cardiomyopathy [18]. As this condition is similar to that observed in Keshan disease, it appears that TR3 reduction in heart tissue must play a major role in the development of this disorder as well as that observed in the corresponding mouse model. Interestingly, GPx1 knockout mice have greater susceptibility to infection with coxsackie B3 virus than wild type mice [51] and to doxorubicin-induced cardiotoxicity [52] providing further evidence of the roles of selenoproteins in proper cardiac function. The targeted removal of *Trsp* in endothelial cells resulted in embryonic death, whereas *Trsp* loss in myocytes resulted in no apparent phenotype until about day 12 after birth when the animals apparently suffered cardiac arrest followed shortly thereafter by death [53]. At day 14.5, embryos lacking *Trsp* in their endothelial cells had various abnormalities that included subcutaneous hemorrhaging and erythrocyte immaturity, while histopathology of the selenoprotein-deficient mice showed moderate to severe myocarditis [53]. These data also suggest a direct connection between selenoprotein loss and cardiovascular disease. 

### 2.3. Trsp transgenic/standard or transgenic/conditional knockout mouse models

The fact that the targeted removal of *Trsp* is embryonic lethal [11,27] and that we had generated wild type and mutant *Trsp* transgenic mice [9] afforded us an opportunity to rescue the knockout mouse with mutant or wild type transgenes [10]. Rescue of knockout mouse (Δ*Trsp*) with a transgenic mouse carrying 20 copies of a *Trsp* transgene resulted in a dramatic enrichment of the Sec tRNA^[Ser]Sec^ population and in little or no change in selenoprotein expression in the various tissues examined [10]. This study provided additional evidence that the Sec tRNA^[Ser]Sec^ population is not limiting in selenoprotein synthesis. It is also of interest to note that the number of transgenes encoded in a genome is directly proportional to the amount of tRNA product generated from the transgene at least up to 10 copies [54]. Clearly, the Sec tRNA^[Ser]Sec^ population increases with each additional transgene copy, even up to 40 in number [9], but it is not known at what point the direct proportionality between transgene number and tRNA product level begins to diminish. 

Rescue of Δ*Trsp* with the G37 mutant transgene resulted in dramatic changes in the Sec tRNA^[Ser]Sec^ population and in the subsequent expression of primarily stress-related selenoproteins [10]. The Sec tRNA^[Ser]Sec^ population consisted of a single tRNA isoform that lacked i^6^A and Um34 and housekeeping selenoproteins were expressed, but the expression of stress-related selenoproteins was dramatically reduced [10]. The Δ*Trsp*-G37 transgenic mouse was phenotypically similar to the corresponding Δ*Trsp-Trsp* transgenic mouse with the major difference being that the G37 rescued mouse had reduced fertility in males due to abnormal sperm morphology and reduced litter size in females. The latter study involving the rescue of Δ*Trsp* mice with the G37 mutant transgene provided evidence that stress-related selenoprotein expression is dependent on the mcm^5^Um-containing isoform. However, this study did not unequivocally demonstrate that the observed dependency was due solely to the addition of Um34 to mcm^5^U, because i^6^A was also missing from the G37 mutant tRNA^[Ser]Sec^ [10]. The fact that the synthesis of Um34 to form mcm^5^Um is the critical step resulting in stress-related selenoprotein synthesis was demonstrated by generating the A34 mutant tRNA^[Ser]Sec^ as is further discussed below. 

Targeting the removal of *Trsp* in specific tissues or organs and replacing the Sec tRNA^[Ser]Sec^ population with mutant G37 transgenes resulting in housekeeping selenoprotein expression, and reduced stress-related selenoprotein expression, provides a novel mouse model for studying the roles of the two subclasses of selenoproteins in the targeted tissue or organ. As mice survive following the knockout of *Trsp* and subsequent loss of selenoprotein expression in hepatocytes [12], we used this system as a means of switching on housekeeping selenoprotein expression in liver using either G37 or A34 mutant transgenes [28] (Table 3). Interestingly, no more than two A34 mutant transgenes could apparently be used to generate housekeeping selenoprotein synthesis in liver, whereas a much higher number of G37 mutant transgenes could be used to carry out synthesis of this selenoprotein subclass [28]. As noted above, A at position 34 in tRNA is converted to inosine that would result in this Sec tRNA^[Ser]Sec^ isoform being capable of decoding the two Cys codons in addition to the Sec codon. A higher number of transgenes would result in a more enriched mutant tRNA population that presumably would compete more effectively with the endogenous Cys tRNA population resulting in greater misreading and harm to cell function. Alteration of selenoprotein synthesis in liver with either A34 or G37 mutant Sec tRNA yielded similarly expressed selenoproteins [28]. Clearly, the addition of Um34 is the critical step in the expression of stress-related selenoproteins since the mutations in A34 and G37 are located at very different positions, but both mutant tRNAs lack Um34 and cause similar effects on selenoprotein translation.

**Table 3:** Mutant *Trsp* transgenic/standard or transgenic/conditional knockout mouse models*.*

Transgene and Number^a^	Model Description	Major Findings	Reference
G37 (40)	All tissues lack a wild type *Trsp* gene and are rescued with mutant G37 transgenes.	The absence of Um34 plays a major role in the expression of stress-related selenoproteins, but not housekeeping selenoproteins.	[10]
A34 (2); G37 (2, 16)	*Trsp* is removed in liver and the resulting mouse encodes either mutant A34 or G37 transgenes.	Both mutant tRNAs lacked Um34, and both supported expression of housekeeping selenoproteins (e.g., TR1) in liver, but not stress-related proteins (e.g., GPx 1). **Um34 is responsible for synthesis of a select group of selenoproteins, the stress-related selenoproteins, rather than the entire selenoprotein population.**	[28]
A34 (2); G37 (2, 16)	*Trsp* is removed in liver and the resulting mouse encodes either mutant A34 or G37 transgenes.	In *Trsp* mutant mouse lines, the expression of ApoE, as well as genes involved in cholesterol biosynthesis, metabolism and transport were similar to those observed in wild type mice indicating for the first time that housekeeping selenoproteins have a role in regulating lipoprotein biosynthesis and metabolism.	[55]
A34 (2); G37 (2, 16)	*Trsp* is removed in liver and the resulting mouse encodes either mutant A34 or G37 transgenes.	The loss of selenoproteins in liver was compensated for by an enhanced expression of several phase II response genes and their corresponding gene products. The replacement of selenoprotein synthesis in mice carrying mutant *Trsp* transgenes led to normal expression of phase II response genes. **Provides evidence for a functional link between housekeeping selenoproteins and phase II enzymes.**	[78]

^a^ Number of transgenes carried by transgenic mice is shown in parentheses wherein two separate transgenic mouse lines were generated with the G37 transgenic mice carrying 2 and 16 transgenes in rows 2-4.

An analysis of the plasma proteins in liver Δ*Trsp* mice showed that apolipoprotein E (ApoE) levels were enriched and plasma cholesterol levels were also found to be elevated [55]. In addition, an alteration in the expression of genes involved in cholesterol biosynthesis, metabolism and transport was observed. However, restoration of housekeeping selenoproteins in liver Δ*Trsp*-G37 and Δ*Trsp*–A34 transgenic mice demonstrated that each of these altered components in Δ*Trsp* liver mice was now very similar to the corresponding levels in wild type mice. These data correlated with previous studies showing that Se deficiency resulted in enriched levels of ApoE and the data provided the first indication that housekeeping selenoproteins play a role in regulating lipoprotein biosynthesis and metabolism [55]. 

### 2.4. Other mouse models involving Trsp

Two mouse models involving primarily one of the upstream regulatory regions of *Trsp*, designated the distal sequence element (*DSE*), have been generated [56,57]. It should first be noted that the regulatory regions governing *Trsp* expression are unique among tRNA genes. That is, transcription of *Trsp* is governed by three upstream regulatory sites which are the TATA box motif located at approximately -30, the proximal sequence element (*PSE*) at approximately -70 [58,59,60,61] and the *DSE* at approximately -200 [60]. The *DSE* consists of an activator region (*AE*) containing a SPH motif and an octomer sequence [60]. A transcription factor, designated Sec tRNA gene transcription activating factor (STAF), binds to the *AE* and stimulates Sec tRNA^[Ser]Sec^ transcript expression [62,63,64,65]. STAF is known to have multiple roles in the expression of numerous other genes transcribed by RNA polymerases II and III [66,67,68,69]. The *Trsp* regulatory region has been reviewed in detail elsewhere [70]. In one of the two mouse models disrupting the *DSE* to further characterize the role of selenoproteins in development, a 3.2 kb fragment was inserted between the *PSE* and *DSE* which was embryonic lethal due to a loss in *Trsp* transcription and severe reduction in *Trsp* transcripts [56]. The insertion sequence was removable by recombination with *Cre*-recombinase which restored normal levels of *Trsp* transcription. Heterozygous animals encoding the inserted sequence and wild type *Trsp* alleles demonstrated that the enhancer activity of the *DSE* region was tissue dependent wherein heart did not require both wild type alleles for normal *Trsp* expression, but other tissues, including liver, were dependent on both wild type *DSE* alleles.

In another mouse model examining the role of the *DSE* region in *Trsp* transcription, the STAF binding site or *AE*, was removed [57]. Transgenic mice lacking *AE* were generated wherein the mice were also Δ*Trsp* and thus dependent on the mutant transgene for survival. Transcription levels were unaffected or even slightly elevated in heart and testes, but manifested a dramatic reduction in other tissues examined. For example, an approximately 60% reduction was observed in kidney and liver, approximately 70% in spleen and lung and approximately 80% in brain and skeletal muscle. Interestingly, the ratios of the two Sec tRNA^[Ser]Sec^ isoforms, containing either mcm^5^U or mcm^5^Um, were changed significantly and the mcm^5^Um-containing isoform was substantially reduced in all tissues examined. Affected mice manifested a neurological phenotype that was very similar to mice lacking the selenoprotein P gene [23,24,71]. Both *AE*^-^ mice and selenoprotein P deficient mice phenotypically manifested growth retardation, tissue calcification, small spleens as well as liver and brain defects. Selenoprotein synthesis in *AE^-^* mice was most affected in the tissues and organs in which the Sec tRNA^[Ser]Sec^ levels were most severely reduced. Thus, the data strongly suggested that STAF controls selenoprotein synthesis by increasing *Trsp* transcription in an organ-specific manner and by regulating Sec tRNA^[Ser]Sec^ modification. 

## 3. Conclusions

Several mouse models have been designed that use an alteration in *Trsp* expression that in turn alters selenoprotein synthesis to elucidate the roles of this protein class. Transgenic mutant Sec tRNA^[Ser]Sec^ mouse models have been prepared, either in a background of wild type *Trsp* or a background of Δ*Trsp* to demonstrate that the two tRNA^[Ser]Sec ^isoforms, containing either mcm^5^U or mcm^5^Um, are involved in specifically synthesizing housekeeping or stress-related selenoproteins, respectively. Furthermore, these transgenic mice have been used to demonstrate that stress-related selenoproteins have a role in a number of health benefits including preventing certain forms of cancer. The conditional *Trsp* knockout mice have been used to show that selenoproteins serve a wide variety of roles in development and health including endothelial cells, the immune system, and heart, brain and skin function and development. Furthermore, the *Trsp* conditional knockout mice have been used to develop a model for Kashin-Beck disease [48]. 

Although mouse models involving Se-containing proteins, both those altering selenoprotein expression by altering Sec tRNA^[Ser]Sec^ expression, as described herein, and those altering individual selenoprotein expression by altering the corresponding gene expression, as thoroughly reviewed elsewhere [26], have aided in advancing our understanding about the intracellular roles of selenoproteins, there is still much to be done. Many biochemical and other studies involving individual selenoproteins, including their targeted removal using RNAi technology, have also elucidated our understanding of the functions and characteristics of selenoproteins (see reviews in [36,37]). However, the functions of only about one-half the total number of selenoproteins arising from the 24 known selenoprotein genes in rodents and 25 in humans [8] are known. Of the many health benefits attributed to Se through the years (see Introduction), it is highly significant to note that as more and more has been learned about the functions of selenoproteins, we can now appreciate the fact that this class of proteins is responsible for many of these health benefits. Most certainly, as we understand the functions, interactions and interplay of more selenoproteins, many more roles of these Se-containing proteins in health and development will come to light. 
